# Maternal near-miss and mortality in Sayaboury Province, Lao PDR

**DOI:** 10.1186/1471-2458-14-945

**Published:** 2014-09-12

**Authors:** Phadouangdeth Luexay, Laopaiboon Malinee, Lumbiganon Pisake, Bouvier-Colle Marie-Hélène

**Affiliations:** Department of Public Health, Sayaboury Province, Lao PDR, Laos; Department of Biostatistics and Demography, Faculty of Public Health, Khon Kaen University, 123 Mittapharp Road, 40002 Muaeng district, Khon Kaen, Thailand; Department of Obstetrics and Gynaecology, Faculty of Medicine, Khon Kaen University, Khon Kaen, Thailand; INSERM-Unité 953 Recherche épidémiologique en santé périnatale et santé des femmes et des enfants, 53 av de l’Observatoire, Maternité de Port Royal, 75014 PARIS, 33-1-42 34 55 72 France

**Keywords:** Maternal near- miss, Maternal mortality, Severe maternal outcome, Maternal complication

## Abstract

**Background:**

Maternal near-miss (MNM) incidence is the indicator reflecting maternal healthcare services. This study aimed to determine the burden of maternal near-miss and maternal deaths in Sayaboury Province, Lao PDR.

**Methods:**

A descriptive study was done in a cohort of 1215 pregnant women, who had their last normal menstrual period (LMP) between 1 August and 31 December, 2010. WHO criteria for MNM were used to identify near-miss cases and maternal deaths during February – November 2011. Data of maternal characteristics, MNM, and maternal deaths were prospectively collected by primary health care workers in the villages under supervision of health staff in local health centers and by the head nurses of the gynecology - obstetric wards in the studied hospitals. Frequencies with 95% confidence intervals (CIs) were used to describe maternal near-misses and maternal deaths.

**Results:**

Overall, 92.5% of the 1215 pregnancies were delivered, 7.5% were aborted. Eleven women were identified as near-miss cases, giving a maternal near miss (MNM) ratio of 9.8 (95% CI: 4.9 -17.5)/1,000 live births. With two maternal deaths, the maternal mortality ratio (MMR) was 178 (95% CI: 50–650)/100,000 live births. Together, these constituted 13 cases of severe maternal outcome (SMO) and given the SMO ratio of 11.6 (95% CI: 6.2 - 19.8)/1,000 live births.

**Conclusion:**

The study shows a surprisingly low MNM ratio and MMR in Sayaboury Province, Lao PDR. Generalization of the results is limited by problems in applying standard criteria for the identification of near-misses in the communities and local hospitals. However, the findings are considered to have important implications for the improvement of maternal health services in low resource settings, e.g. to obtain valid and reliable maternal near miss and maternal deaths for the whole country.

## Background

The fifth Millennium Development Goal (MDG) is to improve maternal health. The two main targets for improvement are 1) to reduce the maternal mortality ratio(MMR) by three quarters between 1990 & 2015 and 2) to achieve universal access to reproductive health by 2015 [1, 2]. Levels of maternal mortality have decreased, but the global maternal mortality ratio declined by only 2.3% per year between 1990 and 2008. This figure is far from what is required to achieve MDG 5 with the aim of an annual decline of 5.5% [1–3].

In spite of the efforts of international developments in maternal health care, maternal deaths and disabilities remain a major public health problem in developing countries. Lack of reliable and up-to-date statistics on maternal deaths also remains a major challenge to the implementation of a master plan in the developing countries to accelerate achieving the Millennium Development Goal related to maternal health (MDG-5) [2–4]. There are 14 countries in the world which have MMR of at least 1,000 per 100,000 live births. In most developing countries where there is no comprehensive registration of deaths, reliability of maternal mortality ratios is still questionable because of lack of medical certification of death [1, 4].

Maternal mortality is the health indicator which shows the greatest gap between the rich and poor countries. Whereas nine maternal deaths per 100,000 live births have been reported in developed countries, 450 maternal deaths per 100,000 live births occurred in developing countries. Approximately 13,000 maternal deaths occur annually in the Western Pacific Region, with large differences within developing countries (urban–rural, rich-poor) [5]. By region, the MMR is highest in Africa (830) and some countries of the Western Pacific Region, the Middle East, followed by Asia (330), Oceania (240), Latin America and the Caribbean (190), and is lowest in the developed countries [1, 6, 7]. In the Lao People’s Democratic Republic, the MMR has been roughly estimated to be 400 – 600 for every 100,000 live births [4, 8–10]. When compared with neighboring countries in South - East Asia, the maternal health indicators for Lao PDR are the worst in the region [5, 8].

However, despite the high maternal morbidity ratios in many resource-poor settings, maternal deaths are still rare in absolute numbers [6]. The study of women who survive life-threatening complications related to pregnancy, called maternal near-miss cases, may represent a practical alternative to surveillance of maternal morbidity and mortality [11]. There have been no official reports of maternal near-miss in the Lao PDR. The Ministry of Health (MOH) of Lao PDR, in cooperation with international agencies, has recently made serious efforts to improve maternal and child health. They have attempted to reduce maternal mortality through implementing new or additional maternal and child health (MCH) service-related activities, such as trained traditional birth attendances (TBA) at the village, trained skill birth attendances (SBA) in the health centers, supported family planning and EPI etc. Despite the efforts, maternal health status was still relatively poor in some areas of the country, and major capacity strengthening is still required in the areas of poor MCH services for instance in mountainous remote areas. Official statistics on MCH indicators have been improving over the past decades, but they remain below international standards. The lack of reliable and up-to-date statistics on maternal deaths and morbidities remains a major challenge for achieving accelerated progress towards the Millennium Development Goal related to maternal health (MDG-5) [1, 4, 12].

Sayaboury Province is one of the remote, mountainous provinces located in the northwestern Lao PDR. Many villages in the remote areas have limited access to health services during the wet season, especially in the mountainous, remote villages. In its efforts to increase access through village health volunteers and the village revolving drug funds, the provincial health office (PHO) has managed to reach almost all villages in the province to provide primary health care [8, 12]. An annual report of Sayaboury PHO 2009 showed that 39% of pregnant women delivered in the hospital and 61% delivered at home. Acute obstetric complications were problems for the women living in remote areas, where road access was still difficult. The referral system for pregnant women was ill-equipped, especially when they were high risk for developing complications.

To address the quality of maternal healthcare services in a low resource setting, this study was conducted to determine the incidence and causes of maternal near-misses and deaths in Sayaboury Province, Lao PDR.

## Methods

### Population and setting

This was a population-based descriptive study to prospectively detect maternal near-misses and maternal deaths in four randomly selected districts from a total of eleven districts in Sayaboury Province during February – November 2011. Two hundred and forty three villages in these four districts were our study sites. These are small villages with a total population of 199,238 people and only 98,164 women. Pregnant women, who had their last normal menstrual period (LMP) between 1 August and 31 December, 2010, were recruited. The LMP range was chosen intending to have enough pregnant women based on the estimated sample size and to follow up the eligible women for their pregnancy outcomes (especially maternal near miss and maternal death). We also had information about those women who aborted before we started data collection from the records routinely collected by traditional birth attendants (TBAs). Exclusion criteria were women who planned to move out of the area during the study period. The eligible women were identified by trained village health volunteers (VHVs) and TBAs under the supervision of health center staff. In cases of uncertainty, pregnancy was confirmed by a urine dipstick test. Recruited pregnant women were home visited daily to detect complication by trained TBAs and VHVs, who lived in the same villages, over the study period. These were not antenatal care visits. They notified health center staff when pregnant women in their villages had abortion or delivery at home. If pregnant women with severe complications (such as severe haemorrhage, dystocia, hypertension etc.) were notified in the villages by trained TBAs or VHVs, the health center staff from the nearest health center went to investigate and confirm the diagnosis of a MNM using case record form (CRF) and referred serious cases to the nearest hospital, which on average was five to ten kilometers away from the village. However in mountainous areas it might take longer time to refer patients to the hospital.

### Variables

The primary outcomes were maternal near-misses and maternal deaths. A maternal near-miss (MNM) was a woman who nearly died but survived a complication, which occurred during pregnancy, childbirth or within 42 days of termination of pregnancy. The modified WHO criteria of organ system dysfunctions [13] as shown in Table 1 were used for the identification of near-misses. Severe maternal outcomes (SMO) were also measured and defined as women who had problem with maternal death or maternal near-miss up to 42 days after giving birth or having an abortion irrespective of gestational age or delivery status.

**Table 1:** **WHO criteria and modified WHO criteria of organ system dysfunctions for detection of maternal near miss (MNM)**

WHO Criteria for MNM detection	Modified WHO criteria for MNM detection at hospital	Modified WHO criteria for MNM detection at community
**1) Cardiovascular dysfunction**	**1) Cardiovascular dysfunction**	**1) Cardiovascular dysfunction**
Shock	Shock	Shock
Cardiac arrest	Cardiac Arrest	Cardiac Arrest
Severe hypoperfusion (lactate >5 mmol/L or >45mg/dL)	Severe hypoperfusion (lactate >5 mmol/L or >45 mg/dL)	
Severe acidosis (pH<7.1)	Severe acidosis (pH<7.1)	
Use of continuous vasoactive drugs	Use of continuous vasoactive drugs	
Cardio-pulmonary resuscitation	Cardio-pulmonary resuscitation	
**2) Respiratory dysfunction**	**2) Respiratory dysfunction**	**2) Respiratory dysfunction**
Acute cyanosis	Acute cyanosis	Acute cyanosis
Gasping	Gasping	Severe tachypnea (respiratory rate>40 breaths per minute)
Severe tachypnea (respiratory rate>40 breaths per minute)	Severe tachypnea (respiratory rate>40 breaths per minute)	
Severe bradypnea (respiratory rate<6 breaths per minute)	Severe bradypnea (respiratory rate<6 breaths per minute)	Severe bradypnea (respiratory rate<6 breaths per minute)
Severe hypoxemia	Severe hypoxemia	
(O2 saturation <90% for _60 min or PAO2/FiO2<200)	(O2 saturation <90% for _60min or PAO2/FiO2<200)	
Intubation and ventilation not related to anaesthesia	Intubation and ventilation not related to anaesthesia	
**3) Renal dysfunction**	**3) Renal dysfunction**	**3) Renal dysfunction**
Oliguria non responsive to fluids or diuretics	Oliguria non responsive to fluids or diuretics	Oliguria non responsive to fluids or diuretics
Severe acute azotemia	Severe acute azotemia	
(creatinine >300umol/ml or >3.5 mg/dL)	(creatinine >300umol/ml or >3.5mg/dL)	
Dialysis for acute renal failure	Dialysis for acute renal failure	
**4) Coagulation dysfunction**	**4) Coagulation dysfunction**	**4) Coagulation/Hematologic dysfunction**
Failure to form clots	Failure to form clots	Excessive bleeding per vagina
Severe acute thrombocytopenia	Severe acute thrombocytopenia	
(<50,000 platelets/ml)	(<50,000 platelets/ml)	
Massive transfusion of blood or red cells (≥5 units)	Massive transfusion of blood or red cells (≥5 units)	
**5) Hepatic dysfunction**	**5) Hepatic dysfunction**	**5) Hepatic dysfunction**
Jaundice in the presence of pre-eclampsia	Jaundice in the presence of pre-eclampsia	Jaundice in the presence of pre-eclampsia
Severe acute hyperbilirubinemia (bilirubin>100 umol/L or > 6.0 mg/dL)	Severe acute hyperbilirubinemia (bilirubin>100 umol/L or > 6.0 mg/dL)	
**6) Neurologic dysfunction**	**6) Neurologic dysfunction**	**6) Neurologic dysfunction**
Prolonged unconsciousness or coma (lasting >12 hours)	Prolonged unconsciousness or coma (lasting >12 hours)	Prolonged unconsciousness or coma (lasting >12 hours)
Stroke	Stroke	Stroke
Uncontrollable fit/status epilepticus	Uncontrollable fit/status epilepticus	Global paralysis
Global paralysis	Global paralysis	
**7) Uterine dysfunction**	**7) Uterine dysfunction**	
Uterine infection or haemorrhage leading to hysterectomy	Uterine infection or haemorrhage leading to hysterectomy	

### Data collection

During February – November 2011, at communities information of pregnancy outcomes, and clinical criteria for the assessment of organ system dysfunctions, such as severe tachypnea (respiratory rate > 40 breaths per minute) or severe bradypnea (respiratory rate < 6 breaths per minute, etc. were collected by the trained health center staff using the pre-established case record form (CRF). All completed CRFs were sent to the head of the maternal and child health (MCH) district office for rechecking and then forwarded to the provincial MCH division. In cases of abortion, the completed CRFs were sent to the provincial MCH division together with a monthly report. Pregnant women, who were referred to the hospitals because of serious complications or intended to deliver their babies in the district or provincial hospital, were followed up until delivery or termination of pregnancy by responsible staff at the district and provincial hospitals.

At the district and provincial hospitals information of pregnancy outcomes and the assessment of clinical criteria, laboratory-based and management-based criteria of organ system dysfunctions of all pregnant women up to 7th day after delivery were collected by trained head of nurse in the gynecology-obstetric wards, and MCH staff at health center or VHV in the village continued to follow- up these women until 42 days after delivery. The completed CRFs at the district and provincial hospitals were sent to the provincial MCH unit together with a monthly report. Maternal deaths were investigated by the heads of district or provincial MCH divisions using the pre-designed investigation form.

### Statistical analysis

The sample size of 1,100 pregnant women was calculated from the SMO rate of 2.3% estimated from the medical records of 6,643 total deliveries in the maternity services of Sayaboury Province in 2009 with a precision of 40% of the estimate and 95% confidence interval.

Descriptive statistics, such as means and standard deviations for continuous variables, and proportion for categorical variables, were used to describe baseline characteristics. The maternal complications, maternal near-misses (MNMs), maternal deaths (MDs) and severe maternal outcomes (SMOs) were estimated using proportions and their 95% confidence intervals (CIs). The analysis was performed using SPSS version 17.

### Ethical approval and participant informed consent

The study was approved by the declaration of personnel and organization department, Ministry of Health (MOH) of Lao PDR. N° 253 on 5 February 2011, declaration of Sayaboury provincial health office [PHO] N° 290, and Khon Kaen University Ethics Committee for Human Research (HE542067)*.* This research is part of PhD study on maternal near-miss and maternal mortality ratio in Sayaboury Province, Lao PDR. All participants were written informed consent for participation in the study. Pregnant women under 18 years old were signed informed consent by their parent.

## Results

**Figure 1:**
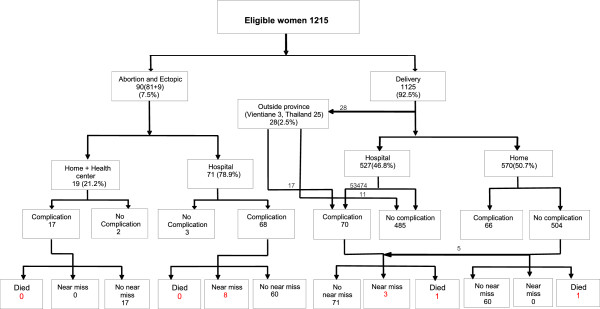
**Flow chart of outcomes followed-up of study participants.**

During the study period, 1215 eligible pregnant women were recruited and followed up until 42 days after delivery or termination of pregnancy in the four randomly selected districts of Sayaboury Province, Lao PDR. There were 81cases of abortion, 9 cases of ectopic pregnancy (7.5%), and 1125 deliveries (92.5%). Five hundred and fifty-five women (49.3%) were delivered in the hospitals, with 527 of these deliveries (46.8%; 527/1125) occurring in the hospitals in Sayaboury Province and 28 (2.5%; 28/1125) in hospitals outside the province (Vientiane and Thailand). Complications were identified in 221 women of whom 68 occurred before 22 weeks of gestation. Among the 221 women, eleven were MNMs and two were maternal deaths. Eight from the eleven MNMs occurred before 22 weeks of gestation (Figure 1).

Table 2 summarizes the baseline characteristics of the 1215 recruited pregnant women. About one-third of the women (38.7%) lived in Sayaboury District. The mean age was 24.4 years (SD 5.6 years) with 11.5% under 19 years of age. Their mean height was 153.4 cm (SD 5.7 cm) and mean weight was 53.0 kg (SD 7.0 kg). The large majority (78%) described themselves as Buddhists. Nine percent had no formal education, and only 2.5% were educated higher than high school level. Most of the women (56.7%) worked as farmers or laborers. Forty three percent of these women were primiparous. 54.5% were institutional deliveries while 43.2% were home deliveries. The largest ethnic group were Lao (70.6%) and 18.3% were from hill tribes (Kamou, Mong, Mien and Pray).

**Table 2:** **Characteristics of pregnant women in the study (N= 1215)**

Characteristics	Number (%)
**Districts**	
Xienghorn	159 (13.1)
Sayaboury	470 (38.7)
Park lay	347 (28.6)
Kaenthao	239 (19.6)
**Age (years)**	
< 19	140 (11.5)
19-34	992 (81.7)
≥ 35	83 (6.8 )
Mean (±SD)	24.4 (±5.6)
**Height (cm)**	
< 145	46 (3.7)
145-160	1065 (87.5)
161-170	104 (8.5)
Mean (±SD)	153.4 (±5.7)
**Weight (kg)**	
cpr< 40	6 (0.5)
40 – 69	1178 (97.0)
≥ 70	31(2.6)
Mean (±SD)	53.0 (±7.0)
**Religion**	
Buddhist	947 (78)
Catholic	46 (4)
Believer inspirits	222 (18.3)
**Education**	
Illiteracy	109 (9.0)
Primary school	561 (46.2)
Secondary school	409 (33.6)
High school	106 (8.7)
After high school (others)	30 (2.5)
**Occupation**	
Farmer/laborer	689 (56.7)
Housewife	401 (33.0)
Government officer/private	125 (10.3)
**Parity**	
0	525 (43.2)
1-2	441 (36.3)
**≥ 3**	249 (20.5)
Median (range)	2 (2–3)
**Place of delivery**	
Provincial hospital	332 (27.3)
District hospital	266 (21.9)
Health center	64 (5.3)
Village(home)	525 (43.2)
Other places	28 (2.3)
**Ethnic background**	
**Living in low land area**	**985 (81.7)**
Lao	858 (70.6)
Lue,	73 (6.0)
Gnouan,	47 (3.9)
Thaidam,	5 (0.4)
Other	2 (0.2)
**Living in hill tribe area**	**230 (18.3)**
Mong	74 (6.1)
Mien	21 (1.7)
Kamou	115 (9.5)
Pray	20 (1.6)

### Pregnancy outcomes

Nine hundred and twenty of the recruited women (75.7%) delivered at term (37–42 weeks of gestational age). Preterm deliveries (22–36 weeks of gestational age) occurred in 155 women (12.8%) and post term delivery fifty cases (4%). Abortions and ectopic rupture cases happened in 90 women (7.5%). The great majority of the women (97.2%; 1093/1125) had vaginal deliveries. Only 2.8% had Caesarean sections (Table 3). Among these 1125 deliveries, there were 1121 singletons and four twins giving a total of 1129 babies. There were 1123 live births (99.8%), six stillbirths (0.5%), 11 early neonatal deaths (1%) and one late neonatal death (0.1%), Table 4.

**Table 3:** **Pregnancy outcomes**

Outcome	Number (%)
**Gestational age (weeks)**	
< 22 (abortion and Ectopic )	90 (7.4)
22-36	155 (12.8)
37- 42	920 (75.7)
> 42	50 (4.1)
**Abortion**	81(6.7)
**Ectopic rupture**	9 (0.7)
Mode of delivery*	
Vaginal delivery	1093 (97.2)*
Caesarean section	32 (2.8)*

*1125 deliveries.

**Table 4:** **Neonatal outcomes**

Outcome	Number (%)
Total deliveries	1125 (92.5)
Single deliveries	1121 (99.6)
Twin deliveries	4 (0.4)
Birth weight (grams)	
< 2500	34 (3.0)
2500 – 3000	705 (63.0)
> 3000	380 (34.0)
Mean (±SD)	2956.8 (±359.9)
Live birth	1123 (99.8)
Stillbirth	6 (0.5)
Early neonatal death (<7 days)	11 (1)
Late neonatal death ( 7 – 28 days )	1 (0.1)

### Maternal complications

The most common complication was haemorrhage in 110 cases (49.7%) and mainly occurred in postpartum period. They were mostly detected in the hospital. Nine of 110 cases were classified as maternal near-miss. The second most common complication was dystocia in 73 cases (33.0%). Forty five cases of them were multiparous and had prolonged labour more than 12 hours. Twenty eight cases were nulliparous with prolonged labour more than 24 hours. All cases of dystocia were assessed by medical doctor in the hospital but the cases detected in the village were confirmed by MCH health center staff. No cases of this category developed MNM or maternal death. The third most common complication was underlying medical conditions (12.6%), including heart diseases, diabetes mellitus, anemia, hyperthyroidism, etc. and one of these led to maternal death. Hypertensive disorder was seen in 16 cases (7.2%); two became maternal near-misses due to preeclampsia in the third trimester of pregnancy.

From the eleven near-miss cases 8 were detected at provincial hospital and 3 found at district hospitals, none were detected in the community. There were only two maternal deaths, one caused by pre-eclampsia in the provincial hospital. The other one was cardiopathy died during referring the mother to the hospital. These outcomes are shown in Table 5.

**Table 5:** **Frequencies of near misses and maternal deaths by potential threatened conditions**

Conditions	Numbers ^*^	Number of near miss (%)	Number of maternal death (%)
Haemorrhage	110	9 (8.2)	0
Hypertensive disorders	16	2 (12.5)	1(6.3)
Previous medical conditions	28	0	1(3.6)
Dystocia	73	0	0
Other	2	0	0
Total	221	11 (5.0)	2 (0.9)

*One woman could have more than one conditions.

### Organ dysfunction system

Nine maternal near miss-cases had multiple organ dysfunctions, and the common organ dysfunctions were respiratory, cardiovascular and renal dysfunctions. A similar pattern of multiple organ dysfunctions was seen in the two maternal deaths (Table 6).

**Table 6:** **Frequencies of maternal near-miss and maternal death by type of organ systems dysfunction**

Dysfunctional organ	Number of near miss cases	Number of maternal death
Respiratory dysfunction	9	2
Renal dysfunction	-	1
Cardiovascular dysfunction	8	2
Hepatic dysfunction	1	-
Neurologic dysfunction	1	1

### Severe maternal outcomes

There were 13 cases of SMO represent a SMO ratio of 11.6 (95% CI 6.2-19.8)/1,000 live births, and the 11 near-miss cases give a MNM ratio of 9.8 (95% CI 4.9 - 17.5)/1,000 live births). Two maternal deaths represent a maternal mortality ratio (MMR) of 179 (95% CI 50 – 650)/100,000 live births). The mortality index (MI = MD/MNM + MD) was 15.3%, and the maternal near-miss mortality ratio (MNM: MD) was 5.5:1 (Table 7).

**Table 7:** **Maternal near-misses and maternal deaths**

Outcomes	Number	Ratio (95% CI)	Rate***(95% CI)
Severe maternal outcome	13	11.6* (6.2-19.8)	10.7(6.3 -18.2)
Maternal near- miss	11	9.8* (4.9 – 17.5)	9.1 (5.1- 16.2)
Maternald eath	2	178.7** (50 – 650)	
Mortality index	15.3%		
Maternal near-miss mortality ratio (MNM: 1 MD)	5.5: 1		

*per 1000 live births, **per 100,000 live births, ***per 1000 eligible women.

## Discussion

Our results represent an investigation of maternal mortality and maternal near-miss based on the prospective data collection in both community and local hospitals in the Lao PDR. The presence of organ system dysfunction criteria recommended by the WHO (respiratory, cardiovascular, renal, hepatic, neurologic dysfunctions) were used for the identification of near-miss cases. We found MNM ratio of 9.8 (95% CI: 4.9 -17.5) per 1,000 live births, MMR of 179 (95% CI: 50–650) per 100,000 live birth, both of which appear to be surprisingly low.

For the MMR our finding was quite comparable to the recent study conducted in the North-Western Border of Thailand where the MMR was 184 (95% CI:150–230) per 100,000 live births [14]. The other comparable result of MMR of 158 per 1,000 live births was reported in the study of maternal mortality estimation at the sub-national level in Bangladesh [15]. Both studies were done in Asian countries similar to Lao PDR.

However, our finding was much lower than those reported in previous studies conducted in other developing countries in other regions. One population based survey of maternal mortality in West Africa found MMR of 311 per 1,000 live births [16]. Another retrospective hospital based study found the MMR of 432 per 1,000 live births in a rural hospital in Sudan [17]. The present study was population based, with prospective data collection in both the community and local hospitals. Retrospective data collection in a central or general hospital (tertiary care) in the previous studies would give higher numbers of maternal death, because of differences in the study population; mothers who gave birth in those hospitals came from unlimited catchment areas and higher proportion of complicated cases [16, 17].

To some extent, our findings can be explained in the context of the present conditions of MCH and other health services in Sayaboury Province, where the environment was different from the previous studies. Altogether, 629 village health volunteers and 573 traditional birth attendants have been trained in maternal and child care for all villages, and they are regularly supervised and attend refresher training [12]. In addition, the health system has been receiving both technical and financial support from international agencies since 1984for the MCH services at all levels. Our findings are consistent with the trend in MMR of Lao PDR reported from 1990 to 2010 by WHO, et al. [18]. The estimates MMR per 100,000 live births were 1,600 in 1990 and 470 in 2010 with a 70% reduction between the two years. The trend is progress towards improving maternal health according to the MDG 5 [18].

Although there were difficulties in transferring patients to the provincial hospital during the wet season, the geography of the province is such that all its districts share a border with Thailand and some with Vientiane Province. When an emergency occurs, there were options for seeking care in a neighboring country or in Vientiane Capital City. The above reasons may account for the low maternal mortality found in our study compared to those suggested by the national statistics [4, 5] and previous studies in developing countries [11, 16, 17].

The present study also found a maternal near-miss ratio of only 9.8 per1, 000 live births which was much lower than those in previous studies. Souza et al. reported the MNM ratio of 34.3 per 1,000 live births in the WHO global survey on maternal and perinatal health done in 120 of eight Latin American countries [7]. Almerie et al. reviewed cases of maternal near- miss and maternal mortality in a maternity university hospital, Damascus, in Syria [6] and reported the MNM ratio of 32.9 per 1,000 live births. A prospective study in Ghana showed the MNM ratio of 28.6 per 1000 live births in a teaching Hospital (KBTH) [19]. Ali et al. reported the MNM ratio of 21.1 per 1,000 live births in a rural hospital in Sudan [17]. The reasons for our lower ratio may be because the previous studies were hospital-based and tend to have higher ratios because of the higher proportion of complicated cases than our community-based study. However our result was quite similar to the report of the WHO Multicounty Survey on Maternal and Newborn Health [20] which presented the ratio of 8.3 per 1, 000 live births. In addition, it was little higher than that of Jabir et al. reported the ratio of 5.06 per 1,000 live births in Baghdad, Iraq [21].

The rates of maternal near-miss may differ when using different identification criteria. A one year retrospective chart review study of medical records of 1,163 obstetric hospital admissions of a tertiary maternity hospital in Rio de Janeiro, Brazil was conducted in 2013 [22]. It underlined that different approaches entail different estimates of MNM. The maternal near-miss ratio was 27 per 1,000 live births using WHO maternal near-miss criteria compared with 123 per 1,000 live births of using the Waterston criteria. Furthermore a systematic review of the prevalence of maternal near miss by Tunçalp, et al. [23] reported the rates of maternal near-miss varied between 0.04% and 4.54% for management-based criteria and between 0.14% and 0.92% for organ-based dysfunction based criteria. The prevalence using different criteria were higher in low-income and middle-income countries of Latin America and Africa [22, 23].

Our finding of maternal near-miss detected may be underestimated. There are several possible reasons for this. Firstly, maternal near-miss was a new marker of health and obstetric care in our setting. The local co-coordinators (VHV, TBA, MCH health center staff) might have difficulties in understanding the concept and process of MNM identification and led to miss some MNM in the communities. Secondly, limitation of the MCH staff and facilities at the district hospitals might be the important factors that did not allow investigators to apply full WHO criteria to detect a maternal near-miss. This was due to a number of factors: (a) the laboratories at the district hospitals had a limited capacity to confirm near-miss cases; (b) the supply of donated blood was inadequate; in the four study hospitals only the provincial hospital had a blood bank, although two district hospitals did have blood stores; and (c) there was a lack of medical equipment and alternative facilities for the care of severely ill patients at the district hospitals. Only the provincial hospital had an intensive care unit. As a consequence, some cases were probably not classified as near-misses because the severity of their condition was unable to be confirmed by the existing facilities.

One significant finding in this study is the very high proportion (7 from 11) of MNMs occurred early in pregnancy, 2 cases related to abortion complication and 5 caused of ectopic pregnancy. Maternal and child health care providers and policy makers should be informed about this important cause of MNM.

They were 9% (8/90 abortions) of all abortion and ectopic pregnancies. This figure was lower than that of 11.1% (61/549 abortions) found in a surveillance network study to identify severe maternal complications associated with abortion in 27 referral obstetrics units across regions of Brazil [24].

Requirement of the complexity of care could be estimated from the MNM incidence and SMO ratio. The higher values, the greater the extent to which women require high complexity care Our findings show the mortality index (MI) was 15% compared to 8.5%of Cecatti et al. who evaluated maternal near miss using WHO criteria and maternal death related to organ failure in Brazil [25]. van den Akker et al. reported a MI of 12% in their study evaluating maternal near miss using organ-failure based criteria as a tool in Southern Malawi [26].The mortality index (MI) represents an estimate of performance. It is recommended that an institute with high index (>20%) may have inadequate quality of obstetric care for the severe cases [24]. Although we had small maternal index in our setting, we could not be sure that there is adequate quality of obstetric care for the severe cases in Sayabury Province. There is lack of resources for obstetric care, such as blood bank, operation room, etc. in many district hospitals of the province. The maternal near-miss: mortality ratio (MNM: 1 MD) was 5.5:1.The ratio can provide direct information on problems related to care of severity obstetric complication and could be used as marker of obstetric care [24].

### Strength of the study

This was the first population-based descriptive study involving a follow-up of pregnant women since the date of their last menstrual period until within 42 days after termination of pregnancy in order to identifying the incidence of MNM and MD in both the community and local hospitals in Lao PDR. The WHO clinical criteria of organ systems dysfunction were used for the identification of near-miss cases, allowing potential international comparisons. Its main strengths were the use of trained PHC workers (TBAs and VHVs) and health center staff to regularly screen eligible pregnant women in the villages and carry out the follow-up of complication cases to detect maternal near-misses and record maternal mortality. The early detection of maternal complication cases in the community and their referral to hospitals for prompt treatment were used to the maximum possible extent to reduce the number of maternal deaths. The follow-up model used in our study is expected to have implications, which will be benefit to women in developing countries.

### Limitations

The study was designed to identify near-misses and maternal deaths in both the community and the local hospitals. However, the WHO standard criteria for the identification of maternal near-misses could not be used in the community, TBAs in the village and local coordinator at the health centers might have limited knowledge to confirm MNMs. In addition, the district hospitals had limited resources for assessing organ dysfunctions, such as limited supply of blood and laboratory facilities. These factors might cause an underestimation of near-miss rates. The maternal near-misses could have been underestimated by the application of the WHO definition of a near-miss which relies on good laboratory and management-based criteria. These potential limitations are consistent with those presented in study of Cecatti et al. [25]. van den Akker et al. used WHO MNM approach studied at Malawi [26] and the study of Nelissen et al. by applying WHO maternal near-miss criteria in low resources setting in rural hospital Tansania [27].

### Implications

The future development of maternal health services (MHS) should include an upgrading of the capacities of MCH services in local hospitals and ways of improving these services in local health centers so that local MHS staff and midwives are better able to recognize and respond to the important factors associated with SMOs. This study was, in essence, a pilot project for the detection of maternal near-misses and maternal deaths in Lao PDR. Using the lessons learnt from this study, it is proposed that the Lao Ministry of Health should consider a nation-wide study of maternal near-misses, for the future development of maternal health services and also for further health research on MCH data collection in poorly resourced areas, especially in the remote provinces of Lao PDR.

## Conclusion

The study shows a surprisingly low MNR and MMR. This may raise questions about the usefulness of the WHO definition of a near-miss in settings with low resources. However, adapted near-miss criteria for the low resources situation may be benefit to the implications for the developing world where health services are also poorly resourced.

## Authors’ information

LP is a PhD student of Public Health at Khon Kaen University (KKU) Thailand and employed at the department of Public Health in Sayaboury Province Lao PDR. ML is a professor for biostatistics at the department of Biostatistics and Demography, faculty of Public Health, Khon Kaen University (KKU) Thailand. PL is a professor for obstetrics and gynaecology at the department of Obstetrics and Gynaecology, faculty of Medicine, KKU, Thailand. MHB is a professorat INSERM-Unité 953 Recherche épidémiologique en santé périnatale et santé des femmes et des enfants, 53 av de l’Observatoire, Maternité de Port Royal, 75014 PARIS 33-1-42 34 55 72, France.
